# Targeted nanopore sequencing for the identification of *ABCB1* promoter translocations in cancer

**DOI:** 10.1186/s12885-020-07571-0

**Published:** 2020-11-10

**Authors:** Mark S. Williams, Naseer J. Basma, Fabio M. R. Amaral, Gillian Williams, John P. Weightman, Wolfgang Breitwieser, Louisa Nelson, Stephen S. Taylor, Daniel H. Wiseman, Tim C. P. Somervaille

**Affiliations:** 1 Leukaemia Biology Laboratory, Cancer Research UK Manchester Institute, Oglesby Cancer Research Building, The University of Manchester, Manchester, M20 4GJ UK; 2 Molecular Biology Core Facility, Cancer Research UK Manchester Institute, The University of Manchester, Alderley Park, Cheshire, SK10 4TG UK; 3 Division of Cancer Sciences, Faculty of Biology, Medicine and Health, Oglesby Cancer Research Building, The University of Manchester, Manchester, M20 4GJ UK; 4 Epigenetics of Haematopoiesis Group, Oglesby Cancer Research Building, The University of Manchester, Manchester, M20 4GJ UK

**Keywords:** Acute myeloid leukemia, ABCB1, Drug resistance, Promoter translocation, HGSC, Ovarian cancer

## Abstract

**Background:**

Resistance to chemotherapy is the most common cause of treatment failure in acute myeloid leukemia (AML) and the drug efflux pump ABCB1 is a critical mediator. Recent studies have identified promoter translocations as common drivers of high *ABCB1* expression in recurrent, chemotherapy-treated high-grade serous ovarian cancer (HGSC) and breast cancer. These fusions place *ABCB1* under the control of a strong promoter while leaving its open reading frame intact. The mechanisms controlling high *ABCB1* expression in AML are largely unknown. We therefore established an experimental system and analysis pipeline to determine whether promoter translocations account for high *ABCB1* expression in cases of relapsed human AML.

**Methods:**

The human AML cell line THP-1 was used to create a model of chemotherapy resistance in which *ABCB1* expression was driven by a promoter fusion. The THP-1 model was used to establish a targeted nanopore long-read sequencing approach that was then applied to cases of *ABCB1*^high^ HGSC and AML. H3K27Ac ChIP sequencing was used to assess the activity of native promoters in cases of *ABCB1*^high^ AML.

**Results:**

Prolonged in vitro daunorubicin exposure induced activating *ABCB1* promoter translocations in human THP-1 AML cells, similar to those recently described in recurrent high-grade serous ovarian and breast cancers. Targeted nanopore sequencing proved an efficient method for identifying *ABCB1* structural variants in THP-1 AML cells and HGSC; the promoter translocations identified in HGSC were both previously described and novel. In contrast, activating *ABCB1* promoter translocations were not identified in *ABCB1*^high^ AML; instead H3K27Ac ChIP sequencing demonstrated active native promoters in all cases studied.

**Conclusions:**

Despite frequent high level expression of *ABCB1* in relapsed primary AML we found no evidence of *ABCB1* translocations and instead confirmed high-level activity of native *ABCB1* promoters, consistent with endogenous regulation.

**Supplementary Information:**

The online version contains supplementary material available at 10.1186/s12885-020-07571-0.

## Background

High level expression of the drug efflux pump ABCB1 leading to active efflux from blast cells of standard-of-care chemotherapy drugs such as daunorubicin and cytarabine is one of the strongest predictors for induction failure in acute myeloid leukemia (AML) [1]. Interestingly, recent studies have shown promoter translocations to be common drivers of *ABCB1* expression in recurrent, chemotherapy-treated high-grade serous ovarian (HGSC) and breast cancer [2, 3]. As a consequence *ABCB1* is placed under the control of a strong promoter while leaving its open reading frame intact. The treatment-emergent selection of promoter-translocated *ABCB1* variants provides compelling evidence for a central role of drug efflux in chemotherapy resistance, at least in cancers of ovary and breast. By contrast, the cellular mechanisms controlling *ABCB1* expression in AML are largely unknown. Most publicly available genome sequencing data in AML were generated using whole-exome sequencing which does not detect structural variants (SVs) in non-coding sequences [4]. Furthermore, few studies have included samples from relapsed patients previously treated with intensive chemotherapy, raising a question as to whether *ABCB1* promoter SVs might be present in *ABCB1*^high^ AML relapse cases. We therefore established an experimental system and analysis pipeline to determine whether promoter translocations account for high *ABCB1* expression in cases of relapsed human AML.

## Methods

### Cell culture

THP-1 cells were from DSMZ (Braunschweig, Germany) and cultured in RPMI 1640 medium (Sigma Aldrich) supplemented with 2 mM L-Glutamine (Life Technologies, Carlsbad, CA) and 10% fetal bovine serum (Sigma Aldrich). Whilst under drug selection cells were counted and replated every third day. Cell lines were confirmed mycoplasma-free and authenticated by short tandem repeat DNA profiling.

### Reagents

Daunorubicin and verapamil were from Sigma Aldrich (St. Louis, MO); tariquidar was from Generon (Slough, UK). Compounds were resuspended in DMSO (tariquidar) or ddH20 (verapamil and daunorubicin), aliquoted and stored at − 20 °C. Final DMSO concentration was < 0.5% in all experiments.

### Primary human samples

Primary human AML and HGSC samples were from the Manchester Cancer Research Centre Tissue Biobank (approved by the South Manchester Research Ethics Committee). Their use was authorized by the Tissue Biobank’s scientific sub-committee, with the informed consent of donors. For relapsed AML, the Biobank archive was searched for all samples. Where there were too few cryopreserved cells available for potential downstream analyses, or where the percentage of blasts in the cryopreserved sample was less than 80%, cases were excluded. This left 21 separate samples for analysis (Table S1). Three presentation cases, selected at random, were also included for comparison. For ChIP sequencing, selected samples were thawed and immediately crosslinked.

### Processing of primary HGSC samples

Samples for *ABCB1* quantitative PCR and nanopore sequencing were taken from ex vivo models established using primary ascitic fluid samples, as previously described [5]. Briefly, ascites was centrifuged (500×g for 10 min at 4 °C) and cell pellets pooled in HBSS (Life Technologies). Red blood cells were removed using a red blood cell lysis buffer (Miltenyi Biotec) as per the manufacturer’s instructions. Tumour cells were plated into Primaria flasks containing OCMI. All cultures were incubated for 2–4 days at 37 °C in a humidified 5% CO_2_ and 5% O_2_ atmosphere. Media was replaced every 3–4 days. Upon cell attachment, stromal cells were separated from the mixed sample using 0.05% trypsin-EDTA. Once tumour cells reached 95% confluency, cells were passaged using 0.25% Trypsin-EDTA, centrifuged in DMEM containing 20% FBS and re-plated at a 1:2 ratio. For long-term storage, cells were frozen in Bambanker (Wako pure chemical).

### Cell viability assays

5 × 10^3^ cells were plated in each well of a 96-well plate with media containing a serial dilution of daunorubicin. Plates were incubated for 72 h at 37 °C. 20 μl of 140 μg/mL resazurin (Sigma Aldrich) was added to each well. Plates were then incubated for a further 4 h and read using a POLARstar Omega plate reader (BMG Labtech, Aylesbury, UK).

### RNA sequencing and data analysis

Total RNA was extracted from 5 × 10^5^ cells using QIAshredder spin columns and an RNeasy® Plus Micro kit (Qiagen, Manchester, UK). Prior to sequencing RNA integrity was checked using a 2100 Bioanalyzer (Agilent Technologies, Santa Clara, CA). PolyA libraries were prepared using a SureSelect Strand Specific RNA Library Prep kit (Agilent Technologies) and samples were then barcoded and pooled. Sequencing was performed using a NextSeq system (Illumina, San Diego, CA). Two replicates were sequenced for each cell line (THP-1_S and THP-1_R). A single run of 150 bp paired-end sequencing produced a mean of 55.1 M reads per sample. Reads were aligned to the human genome (hg38) using STAR v2.4.2a [6]. DEseq2 was used to calculate FPKM (fragments per kilobase of transcript per million mapped reads) values for each transcript [7].

### Quantitative PCR

cDNA was generated using a High Capacity Reverse Transcription kit (Applied Biosystems, Foster City, CA). qPCR reactions were performed in MicroAmp® optical 384-well reaction plates and analysed using a QuantStudio® 5 PCR system (Applied Biosystems). Reactions were performed in quadruplicate and included primers for β-Actin (ACTB) as a housekeeping gene. Primers were designed using the Universal Probe Library (UPL) Assay Design Center (Roche, Basel, Switzerland) and purchased from Integrated DNA Technologies (Coralville, IA). Raw fluorescence data was converted to CT values using the Thermo Fisher Cloud facility (Waltham, MA) and normalised to ACTB. Primers were (i) *ACTB* (F) ATTGGCAATGAGCGGTTC, (R) GGATGCCACAGGACTCCAT, UPL probe #11 and (ii) *ABCB1* (F) GGAAATTTAGAAGATCTGATGTCAAAC, (R) ACTGTAATAATAGGCATACCTGGTCA, UPL probe #65.

### Chromatin immunoprecipitation (ChIP) and next generation sequencing

ChIP was performed using anti-H3K27Ac (ab4729, Abcam, Cambridge, UK). 10^8^ cells (THP-1) or 10^7^ cells (primary AML samples) were used for each precipitation using the method described by Lee et al. [8]. Briefly, cells were cross-linked with 1% formaldehyde for 10 min at room temperature before the reaction was quenched with 0.125 M glycine. Cell pellets were washed twice with PBS and nuclear lysates sonicated for 6 cycles using a Bioruptor® Pico (Diagenode, Liege, Belgium). 10 μg of antibody bound to 100 μl of magnetic beads (Dynabeads Protein G, Invitrogen, Carlsbad, CA) was added to each sample and immunoprecipitation performed overnight on a rotator at 4 °C and 20 rpm. After five washes with RIPA buffer (50 mM HEPES pH 7.6, 1 mM EDTA, 0.7% Na deoxycholate, 1% NP-40, 0.5 M LiCl), chromatin IP-bound fractions were extracted by incubating for 15 min at 65 °C with elution buffer (50 mM TrisHCl pH 8, 10 mM EDTA, 1% SDS). Crosslinking was then reversed by incubation at 65 °C for 6 h. RNaseA (1 mg/ml) and proteinase K (20 mg/ml) were added to eliminate RNA and protein from the samples. DNA was extracted using phenol:chloroform:isoamyl alcohol and precipitated by adding 2 volumes of ice-cold 100% ethanol, glycogen (20 μg/μl), 200 mM NaCl and freezing at − 80 °C for at least 1 h. Pellets were washed with 70% ethanol and eluted in 50 μl 10 mM TrisHCl pH 8.0.

Libraries were prepared for sequencing using a Microplex Library Preparation Kit (Diagenode). 200-800 bp fragments were selected using AMPure XP beads (Beckman Coulter, Brea, CA). Sequencing was performed using a NextSeq desktop sequencing system (Illumina) with 75 bp, paired-end high output generating 65-80 M (THP-1_S and THP-1_R) and 34-45 M reads per sample (primary AML samples). Reads were aligned to the human genome (hg38) using BWA-MEM v0.7.15 [9]. Read duplicates were removed using Picard v2.1.0. Reads were further filtered using Bedtools v2.25.0 to keep only paired reads that mapped to standard chromosomes and to remove reads with a mapping quality of less than 10. Reads mapped to blacklisted regions defined by ENCODE were then removed using Bedtools (http://mitra.stanford.edu/kundaje).

### 4C sequencing

4C primer sequences and enzyme combinations were selected using the University of Chicago online tool (http://mnlab.uchicago.edu/4Cpd) with co-ordinates from the *ABCB1* promoter active in THP-1_R cells (hg38, chr7:87,598,302-87,601,399). 4C sequencing was performed according to the protocol developed by Splinter et al. [10]. Briefly, 10^7^ cells were cross-linked with 2% formaldehyde for 10 min at room temperature before the reaction was quenched with 0.125 M glycine. Cells were lysed with buffer containing 50 mM Tris–HCl pH 7.5, 150 mM NaCl, 5 mM EDTA, 0.5% NP-40, 1% TX-100 and 1x complete protease inhibitors (Roche, #11245200). The cross-linked nuclear preparation was then incubated with DpnII. Digestion was confirmed by reversing crosslinking for an aliquot and running on a 0.6% agarose gel. Samples were then ligated overnight at 16 °C using T4 DNA ligase (Roche, #799009). Ligation efficiency was again confirmed with 0.6% agarose gel. Crosslinking was reversed and DNA extracted using phenol-chloroform. Samples were then subjected to a second digestion using Csp6I. Ligation was again performed overnight at 16 °C using T4 DNA ligase. DNA was then extracted using phenol-chloroform and purified with a QIAquick PCR purification kit (Qiagen, #28104). PCR primers were designed to incorporate 4C primers with a barcode and Illumina adapter sequences thus:

Reading primer: 5′ P5-Barcode-Primer 3′.

Non-reading primer: 5′ P7-Primer 3′.

Reading: GAGATACCAGGTCTGATC.

Non-reading: AGGGTAGGTATTCCACTTTT.

Illumina adapter sequences:

P5: AATGATACGGCGACCACCGAGATCTACACTCTTTCCCTACACGACGCTCTTCCGA TCT.

P7: CAAGCAGAAGACGGCATACGAGAT.

Non-reading primer: CAAGCAGAAGACGGCATACGAGATAGGGTAGGTATTCCACTTTT.

Reading primer THP-1_S: AATGATACGGCGACCACCGAGATCTACACTCTTTCCCTA CACGACGCTCTTCCGATCTGCCAATGAGATACCAGGTCTGATC (barcode GCCAAT).

Reading primer THP-1_R: AATGATACGGCGACCACCGAGATCTACACTCTTTCCCT.

ACACGACGCTCTTCCGATCTCTTGTAGAGATACCAGGTCTGATC (barcode CTTGTA).

PCR was performed with Expand Long Template Polymerase (Roche, #11759060001) using 3.2 μg of 4C template product and then purified using a High Pure PCR Product Purification Kit (Roche. #11732676001). Library quality was assessed using a 2100 Bioanalyzer (Agilent Technologies). Samples were sequenced with 10% phiX using a MiSeq desktop sequencing system (Illumina) with 75 bp single-end settings generating a mean of 1.4 M reads per sample. Sequencing data was deconvoluted using cutadapt v1.18. Reads were mapped and analysis performed using 4Cseqpipe [11].

### Target enrichment and nanopore long-read sequencing

Genomic DNA was extracted from 5 × 10^6^ cells using a QIAamp DNA mini kit (Qiagen, Manchester, UK). 1 μg of genomic DNA was sheared with a g-TUBE™ (Covaris, Inc., Woburn, MA) using 50 μl sample volume and a 30s spin at 11,000 rpm followed by inversion and a further 30s at 11,000 rpm. Barcoded libraries were prepared from 45 μl of fragmented DNA using a Ligation Sequencing Kit SQK-LSK108 / SQK-LSK109 (Oxford Nanopore Technologies, Oxford, UK) according to the 1D Sequence Capture protocol with the following modifications: the pre hybridisation PCR was replaced by a custom PCR with 6 cycles using Herculase II fusion DNA polymerase (Agilent Technologies). The subsequent AMPure XP purification was performed using a 0.6x bead to sample volume ratio. All purified product was used for the hybridization using an oligonucleotide designed using the online Agilent SureDesign service (https://earray.chem.agilent.com/suredesign/home.htm). The library targeted a 500 kb region that included *ABCB1* and upstream neighbouring genes (*RUNDC3B*, *SLC25A40*, *DBF4* and *ADAM22*; chr7:87,132,949-87,632,948 hg19). Following pull-down, the post-capture amplification was replaced by a custom PCR with 14 cycles as described above. Amplified libraries were quantified by Qubit (Invitrogen) and TapeStation 2200 (Agilent Technologies). An additional amplification was performed using 10 cycles of PCR and the product was quantified. Amplified libraries were again quantified by Qubit and 1 μg of each library used for the End-prep. AMPure XP purification was performed using a 0.47x bead to sample ratio. Four end-prepped libraries were pooled into one adapter ligation reaction using 125 ng of each, as measured by Tapestation. A 0.47x bead to sample ratio was used for the AMPure XP bead binding. Sequencing was performed on the MinION using the R9.4 / 9.4.1 Flow Cell FLO-MIN106 / FLO-MIN106D (Oxford Nanopore Technologies) according to the manufacturer’s instructions. Target enriched DNA from THP-1_R was also subjected to conventional sequencing using a MiSeq desktop sequencing system (Illumina) with 1% phiX and 251 bp, paired-end settings generating 1.92 M reads. Nanopore sequencing of THP-1_R generated 0.92 M reads with a mean read length of 1901 bp and a maximum read length of 224.8 kb. The coverage of the targeted region was 58.1x. Nanopore sequencing of AML/HGSC generated an average of 1.6 million reads per sample (range 0.8–3.0 million) with a mean read length of 1581 bp. FASTQ files were aligned against the GRCh38/hg38 reference human genome using LAST after training the aligner with a subsample of 10,000 reads [12]. Structural variant calling was performed using NanoSV [13]. Structural variants were confirmed through manual inspection and identification of chimeric reads using IGV (https://software.broadinstitute.org/software/igv/).

### Flow cytometry & assessment of daunorubicin retention

Flow cytometry was performed using an LSR II flow cytometer (BD Biosciences, Franklin Lakes, NJ). FlowJo v10.1 (BD Biosciences) was used to analyze data. To assess daunorubicin retention 5 × 10^5^ cells were resuspended in PBS containing 1 μM daunorubicin with 40 μM verapamil or 5 nM tariquidar or vehicle. Samples were incubated for 2 h at 37 °C then placed on ice to prevent further efflux. Daunorubicin accumulation was assessed by flow cytometry.

## Results

In the first instance we sought to establish whether activating promoter translocations could be experimentally induced in AML. *ABCB1* promoter translocations have been described in several solid malignancy cancer cell lines but not in human myeloid leukemia cell lines [14]. We exposed the human AML cell line THP-1 to escalating doses of daunorubicin over 142 days and generated a resistant line (THP-1_R) which exhibited a 28.3-fold increase in daunorubicin IC_50_ (Figs. S1A and S1B). RNA sequencing and confirmatory quantitative PCR indicated that *ABCB1*, which was not expressed in THP-1 cells, was one of the most highly expressed genes in THP-1_R cells (among the top 2.5% of genes by read count; Table S2, Fig. S1C). Parental drug-sensitive THP-1 (THP-1_S) cells did not efflux daunorubicin whereas THP-1_R cells exhibited robust drug efflux that was completely reversed by either verapamil (a non-specific ABC transporter substrate) or tariquidar (a highly specific inhibitor of ABCB1) [15], confirming that efflux was due to ABCB1 (Fig. S1D). Importantly, the resistance of THP-1_R cells to daunorubicin was a stable cellular phenotype and persisted over 3 months of continuous culture without daunorubicin selection, suggesting constitutive expression (Fig. S1E).

**Fig. 1:**
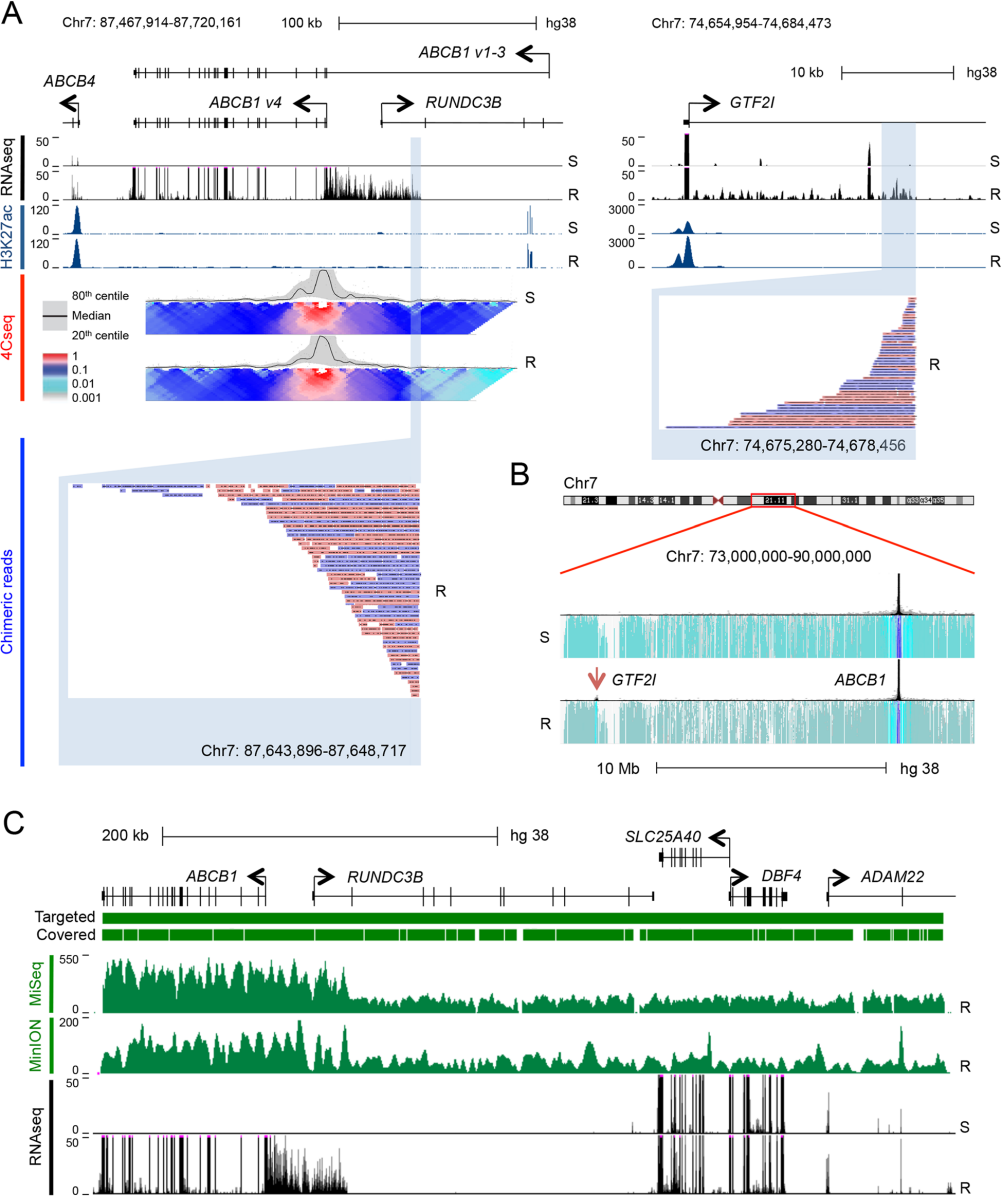
*GTF2I-ABCB1* fusion induced by daunorubicin exposure of THP-1 AML cells identified by nanopore sequencing. **a** RNAseq and H3K27Ac ChIPseq tracks for the indicated cell lines surrounding *ABCB1* (upper left panel) and the promoter of *GTF2I* (upper right panel). Middle left panel: local contact 4Cseq profile of THP-1_S and THP-1_R cells using an *ABCB1* promoter centered viewpoint. Lower panels show chimeric reads generated by targeted nanopore sequencing of the *ABCB1* locus. In each region the breakpoint is highlighted in blue. Pink or blue coloring of reads indicates orientation. **b** 4Cseq contact profile for a 17 Mb region of chromosome 7. **c** Tracks show the 500 kb region surrounding *ABCB1* targeted for sequencing, the region expected to be covered by RNA hybrid capture and the coverage achieved using conventional sequencing (MiSeq) and nanopore long-read sequencing (MinION) of THP-1_R cells. Bottom panel shows RNAseq tracks from THP-1_S and THP-1_R cells

*ABCB1* codes for four protein coding transcript variants and contains two promoters separated by a ~ 110 kb intron that is transcribed in variants 1–3. This large first intron, which overlaps part of the coding sequence for *RUNDC3B* (Fig. 1a), appears to be a common site for promoter translocations. In fact, all of the high-grade serous ovarian cancer samples with *ABCB1* SVs characterised by Christie et al. [3] had at least one SV involving intron 1. We observed that THP-1_R cells expressed *ABCB1* transcript variant 4 but inspection of RNA sequencing tracks revealed additional transcription of intron 1, starting ~ 48 kb upstream of the variant 4 promoter (Fig. 1a). ChIP sequencing for H3K27 acetylation, a histone modification that marks active promoters and enhancers, found no acetylation of either *ABCB1* promoter confirming that neither was active despite high levels of transcription (Fig. 1a). To identify the co-opted promoter we performed 4C-sequencing of THP-1_S and THP-1_R cells with a viewpoint centered on the *ABCB1* variant 4 promoter and observed in resistant cells both an abrupt loss of contacts within *ABCB1* intron 1 and gain of a distant interaction with *GTF2I* (Fig. 1a and b). The *GTF2I* promoter is located ~ 12.94 million base pairs centromeric to the *ABCB1* variant 4 promoter on chromosome 7q; it was strongly acetylated and *GTF2I* expression was among the top 25% of genes in both lines (Fig. 1a and Table S2). These data indicate that a daunorubicin-induced translocation fusing the *GTF2I* promoter with the coding sequence for *ABCB1* was responsible for the acquisition of constitutive high level *ABCB1* expression in THP-1_R cells.

**Fig. 2:**
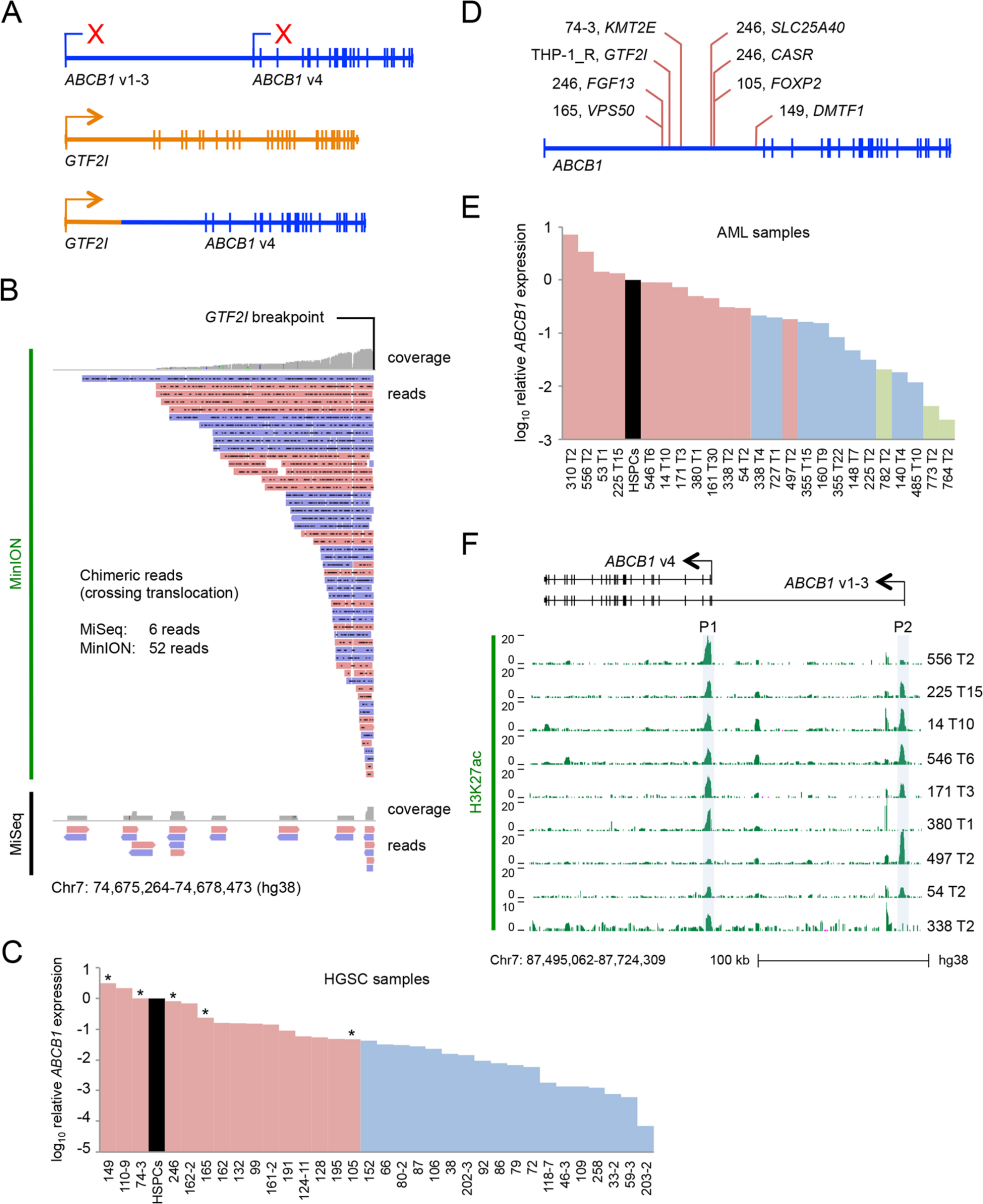
Nanopore sequencing identifies *ABCB1* fusions in *ABCB1*^high^ relapsed primary human HGSC but not primary AML. **a** Image illustrates the *GTF2I-ABCB1* translocation. Red Xs indicate absent transcription from normal *ABCB1* promoters in THP-1_R AML cells. **b** Tracks show the *GTF2I* portion of chimeric reads spanning the *GTF2I-ABCB1* translocation generated by nanopore long-read sequencing (MinION; upper panel) and conventional sequencing (MiSeq; lower panel) of THP-1_R. Pink or blue coloring of reads indicates orientation. **c** *ABCB1* expression levels in primary HGSC samples relative to normal human CD34^+^ haematopoietic stem and progenitor cells (HSPCs), as determined by quantitative PCR. Targeted nanopore sequencing was performed on the samples highlighted in pink; samples with a functional *ABCB1* intron 1 fusion are marked with asterisks. **d** *ABCB1* intron 1 breakpoints identified in HGSC samples & THP-1_R cells are indicated, together with the partner gene. **e** *ABCB1* expression levels relative to normal human CD34^+^ HSPCs as determined by quantitative PCR in primary relapsed AML samples. Targeted nanopore sequencing was performed on the samples highlighted in pink. Three presentation AML samples are also shown in green. **f** H3K27Ac ChIPseq tracks showing the acetylation of *ABCB1* promoters for nine of the relapsed AML samples shown in (E). For (C), (E) and (F) number indicates Biobank identifier; T = timepoint

Having demonstrated the presence of an experimentally initiated *ABCB1*-activating promoter translocation in THP-1_R cells, we developed a long-read sequencing technique to confirm this finding and to facilitate screening of primary patient AML samples for similar SVs. We performed targeted sequencing of a 500 kb region of chromosome 7 that contained both *ABCB1* and *SLC25A40,* which is the most common translocation partner for *ABCB1* in chemotherapy-treated breast and ovarian cancer patients [3]. The region was enriched using a library of biotinylated oligonucleotides that provided 95.5% coverage of the targeted sequence (Fig. 1c). Uniquely mapping oligonucleotides cannot be produced for regions containing repetitive sequences. Given that breakpoints frequently contain repeats we first compared conventional sequencing to long-read nanopore sequencing, which generates reads of up to 15 kb that can bridge translocations involving repetitive sequences (Fig. 1c) [16]. Both approaches identified the *GTF2I* promoter: *ABCB1* translocation (Figs. 1a and 2a). However, despite lower coverage of the targeted region (58.1x versus 368.6x for MiSeq) nanopore long-read sequencing generated 52 chimeric reads that crossed the breakpoint and mapped unambiguously to *GTF2I*, compared with only six by conventional sequencing (Fig. 2b). These data demonstrate that long-read nanopore targeted sequencing efficiently identified the *GTF2I* promoter: *ABCB1* translocation.

In chemotherapy-treated HGSC Christie et al. [3] identified *ABCB1* fusions in 20/108 (18.5%) of cases, increasing to 31.3% if only *ABCB1*^high^ cases were considered. To assess the sensitivity of our nanopore long-read sequencing approach we quantified *ABCB1* expression in 33 samples of metastatic HGSC recovered from ascitic fluid (Fig. 2c), and sequenced the 15 cases with the highest expression (Table S3). In 5/15 cases we identified translocations involving splicing of non-coding 5′ regions of partner genes to exon 2 of *ABCB1* as a result of an intron 1 breakpoint (Table S4 and Fig. 2c-d); in one sample three separate fusion genes were identified. In addition to expanding the repertoire of reported *ABCB1* fusions in HGSC, our data also demonstrate that targeted nanopore sequencing is at least as sensitive as the multimodal approach taken by Christie et al. [3].

We next identified in our Biobank 21 cases of relapsed AML with sufficient cryopreserved cells for downstream analyses, and once more determined *ABCB1* expression levels by quantitative PCR (Fig. 2e). All but two patients had previously been treated with anthracycline-containing chemotherapy and all had initially achieved a complete morphological remission. Patient characteristics and molecular genetic features are shown in Table S1. At original presentation 16/21 cases exhibited a normal karyotype, 2/21 an *MLL* gene rearrangement, 1/21 a t (8;21), 1/21 inv. [16] and 1/21 trisomy 11 (Table S1). Blood samples exhibited high blast cell percentages (median 93.5%, range 82–100%). Targeted nanopore sequencing of 12 cases with high level *ABCB1* expression (Fig. 2e) achieved similar coverage to that achieved in THP-1_R (mean 45.3x, range 16.5–82.3), but failed to identify any functional translocations involving *ABCB1* intron 1 (Figs. S2A and S2B). H3K27Ac ChIP sequencing of nine of 12 of the nanopore long read sequenced samples performed in parallel revealed acetylation of one or both *ABCB1* promoters in every sample, demonstrating that high level *ABCB1* expression in relapsed AML is driven by native promoters (Fig. 2f), at least in the samples evaluated. These results are consistent with our recent finding that *ABCB1* expression in primary AML is highly dynamic and regulated by several active enhancers [17].

## Discussion

While we were able to experimentally induce in a human myeloid leukemia cell line the kind of treatment-emergent promoter-translocated *ABCB1* variant found in chemotherapy-treated breast and ovarian cancers, we were unable to identify similar translocations in primary human relapsed AML samples from patients previously exposed to anthracycline-based chemotherapy. This is despite the strong association of *ABCB1* with treatment failure and the high frequency of balanced translocations targeting a range of genes in AML [1, 18]. Instead, as we have recently demonstrated, *ABCB1* expression appears to be regulated by its native promoters and a range of cooperating enhancer elements [17]. One possible explanation for this difference is the relative genetic stability of AML which has one of the lowest mutation frequencies of any cancer [19]. By contrast ovarian and breast cancers have much higher rates of mutation and more frequent microsatellite and chromosomal instability that predicts for poor outcome [20–22]. The mechanism of activation may also depend on the state of the *ABCB1* promoter in different cancers and their cells of origin. Hematopoietic stem cells normally express *ABCB1* and its regulatory mechanisms appear to be preserved in AML, allowing for dynamic expression and adaptation following treatment [17]. In contrast, in tumours and tissues where the *ABCB1* promoter is silenced, translocation may be the only mechanism by which expression can be induced, favouring the selection of structural variants that replace the silenced promoter. Despite the poor performance in clinical trials of inhibitors of ABCB1, recent findings continue to highlight the central clinical relevance of this drug efflux pump.

## Conclusions

Our findings demonstrate the utility of long-read sequencing as an efficient technique for identifying SVs in human cancers and a promising approach for cost effective medical genetics [23]. Whilst our approach expanded the repertoire of reported *ABCB1* fusions in HGSC, we did not identify similar structural variants in *ABCB1*^high^ AML, where expression appears to driven by native promoters. Further characterisation of disease-specific mechanisms of *ABCB1* regulation are needed to inform novel approaches for overcoming chemotherapy resistance.

## Supplementary Information

**Additional file 1: Table S1.** Clinical details of AML samples used for targeted nanopore sequencing and ChIPseq.

**Additional file 2: Table S2.** Expression values (FPKM) for expressed protein coding genes.

**Additional file 3: Table S3.** Clinical details of HGSC samples used for targeted nanopore sequencing.

**Additional file 4: Table S4.** Translocations involving ABCB1 intron 1 detected by targeted nanopore sequencing of HGSC, AML and THP-1 samples.

**Additional file 5: Figure S1. **Induction of *ABCB1 *in THP-1 AML cells with escalating daunorubicin exposure.

**Additional file 6: Figure S2. **Nanopore sequencing of *ABCB1*^high ^relapsed AML.

## Data Availability

Raw data files are available at the Gene Expression Omnibus with accession number GSE159512**.**

## Abbreviations

ABCB1: ABC sub-family B member 1
ACTB: Actin beta
AML: Acute myeloid leukaemia
cDNA: Complementary DNA
ChIP: Chromatin immunoprecipitation
DMSO: Dimethyl sulfoxide
EDTA: Ethylenediaminetetraacetic acid
FBS: Foetal bovine serum
FPKM: Fragments per kilobase of transcript per million mapped reads
GTF2I: General transcription factor IIi
H3K27ac: Histone H3 lysine 27 acetylation
HGSC: High-grade serous ovarian cancer
HSPC: Haematopoietic stem and progenitor cells
qPCR: Quantitative polymerase chain reaction
RPMI: Roswell Park Memorial Institute
RT-PCR: Real-time polymerase chain reaction
RUNDC3B: RUN domain containing 3B
SLC25A40: Solute carrier family 25 member 40
SV: Structural variant
UPL: Universal Probe Library
