# Supplementary material for: Targeted nanopore sequencing for the identification of ABCB1 promoter translocations in cancer
Source: BMC Cancer. 2020 Nov 10;20:1075. doi: 10.1186/s12885-020-07571-0 (PMC7654162; doi:10.1186/s12885-020-07571-0)
Supplement: Supplementary file 5 — Additional file 5: Figure S1. Induction of ABCB1 in THP-1 AML cells with escalating daunorubicin exposure. [file 12885_2020_7571_MOESM5_ESM.pdf]

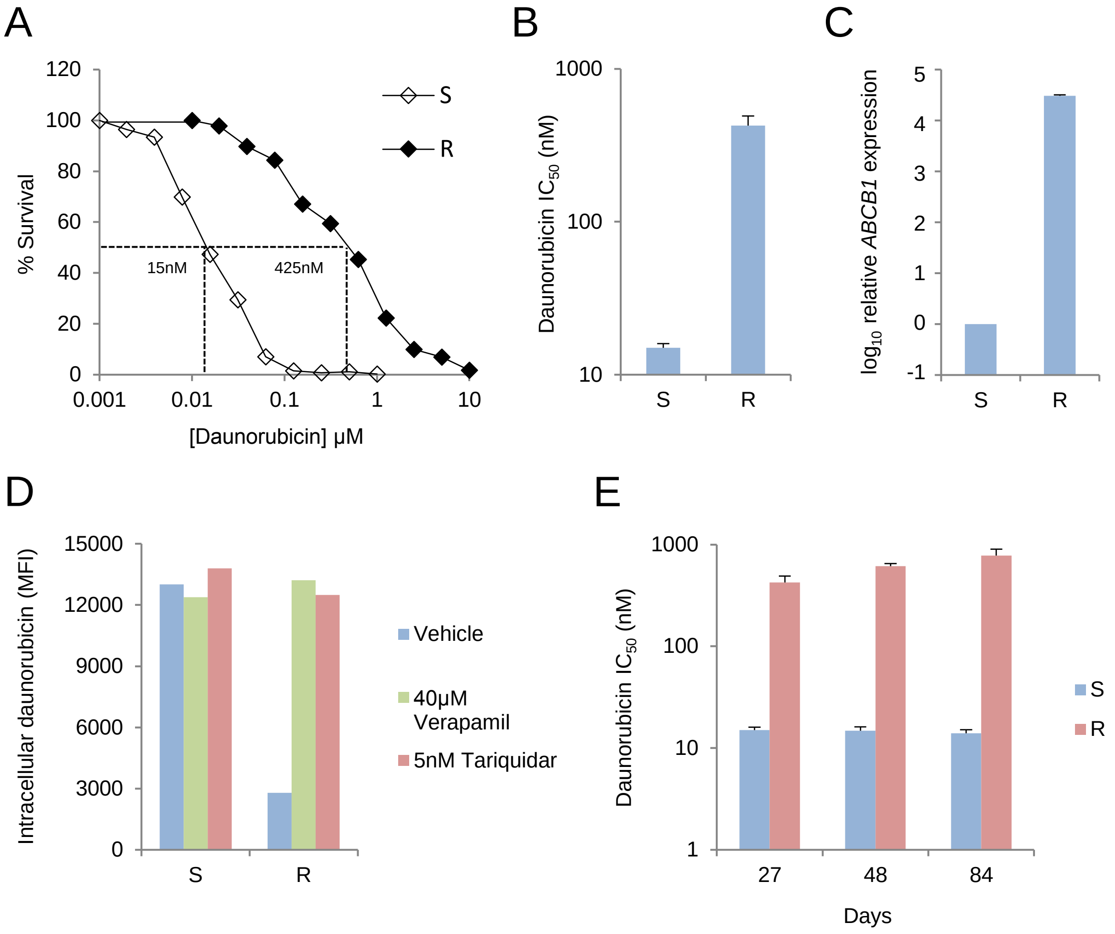

Figure S1. Induction of *ABCB1* in THP-1 AML cells with escalating daunorubicin exposure

(A) Dose response curves for sensitive and resistant lines following 72hr treatment with the indicated dose of daunorubicin. Survival was determined using a resazurin cell viability assay. (B) Bar chart shows mean±SEM IC_{50} values for sensitive and resistant lines (n=4). (C) Mean±SEM fold increase in *ABCB1* expression, as determined by quantitative PCR (n=4). (D) Bar chart shows median fluorescence intensity (MFI) of intracellular daunorubicin following incubation of the indicated cell lines with 1µM daunorubicin in the presence or absence of verapamil 40µM or tariquidar 5nM for 2 hours, as determined by flow cytometry. (E) Bar chart shows mean±SEM IC_{50} values for daunorubicin for THP-1_S or THP1_R cells propagated for the indicated number of days in the absence of daunorubicin (n=4).
