# Supplementary material for: Targeted nanopore sequencing for the identification of ABCB1 promoter translocations in cancer
Source: BMC Cancer. 2020 Nov 10;20:1075. doi: 10.1186/s12885-020-07571-0 (PMC7654162; doi:10.1186/s12885-020-07571-0)
Supplement: Supplementary file 6 — Additional file 6: Figure S2. Nanopore sequencing of ABCB1high relapsed AML. [file 12885_2020_7571_MOESM6_ESM.pdf]

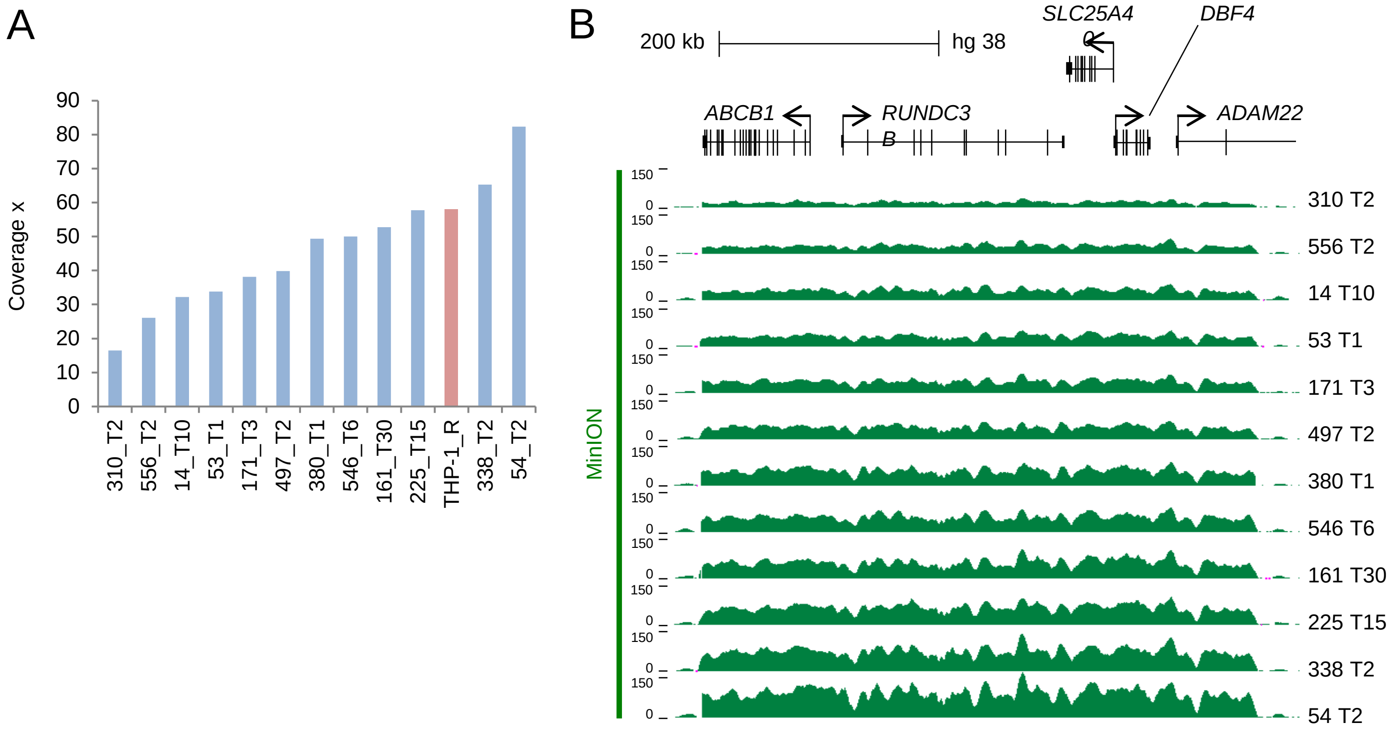

Figure S2. Nanopore sequencing of *ABCB1*^{high} relapsed AML

(A) Bar chart shows coverage of the targeted locus achieved by nanopore sequencing in the indicated patient samples (blue) and THP-1_R (red). (B) Tracks show the 500kb region surrounding *ABCB1* targeted for sequencing and the coverage achieved using long-read nanopore sequencing (MinION) of the indicated *ABCB1*^{high} relapsed AML samples.
